# Porous Silicon as a Platform for Radiation Theranostics Together with a Novel RIB-Based Radiolanthanoid

**DOI:** 10.1155/2019/3728563

**Published:** 2019-03-12

**Authors:** Ulrika Jakobsson, Ermei Mäkilä, Anu J. Airaksinen, Osku Alanen, Asenath Etilé, Ulli Köster, Sanjeev Ranjan, Jarno Salonen, Hélder A. Santos, Kerttuli Helariutta

**Affiliations:** ^1^Department of Chemistry, University of Helsinki, FI-00014 Helsinki, Finland; ^2^Helsinki Institute of Physics, University of Helsinki, FI-00014 Helsinki, Finland; ^3^Department of Physics and Astronomy, University of Turku, FI-20014 Turku, Finland; ^4^Institut Laue-Langevin, 71 Avenue des Martyrs, FI-38042 Grenoble Cedex 9, France; ^5^Drug Research Program, Division of Pharmaceutical Chemistry and Technology, Faculty of Pharmacy, University of Helsinki, FI-00014 Helsinki, Finland; ^6^Helsinki Institute of Life Science (HiLIFE), University of Helsinki, FI-00014 Helsinki, Finland

## Abstract

Mesoporous silicon (PSi) is biocompatible and tailorable material with high potential in drug delivery applications. Here, we report of an evaluation of PSi as a carrier platform for theranostics by delivering a radioactive ion beam- (RIB-) based radioactive lanthanoid into tumors in a mouse model of prostate carcinoma. Thermally hydrocarbonized porous silicon (THCPSi) wafers were implanted with ^159^Dy at the facility for radioactive ion beams ISOLDE located at CERN, and the resulting [^159^Dy]THCPSi was postprocessed into particles. The particles were intratumorally injected into mice bearing prostate cancer xenografts. The stability of the particles was studied *in vivo*, followed by *ex vivo* biodistribution and autoradiographic studies. We showed that the process of producing radionuclide-implanted PSi particles is feasible and that the [^159^Dy]THCPSi particles stay stable and local inside the tumor over seven days. Upon release of ^159^Dy from the particles, the main site of accumulation is in the skeleton, which is in agreement with previous studies on the biodistribution of dysprosium. We conclude that THCPSi particles are a suitable platform together with RIB-based radiolanthanoids for theranostic purposes as they are retained after administration inside the tumor and the radiolanthanoid remains embedded in the THCPSi.

## 1. Introduction

Cancer together with cardiovascular diseases is one of the two most prominent causes for morbidity that burden healthcare systems worldwide [1]. In cancer, the primary tumor is often accompanied by metastases rendering the treatment very complicated. Targeted cancer therapy has, therefore, evolved to provide a mode of therapy with a high precision and broad applicability enabled by extensive studies of tumor dynamics [2]. An appealing technique is to use carrier-mediated targeted delivery of cytotoxic drugs or therapeutic radionuclides [3]. Multifunctional nanoparticles have emerged as a prominent alternative for such carriers, due to the penetrative capabilities enabled by their size and their flexibility toward a multitude of modifications [4, 5].

When loaded with radionuclides, the therapeutic particles can be traced during the treatment through the translational techniques of positron emission tomography (PET) or single-photon emission computed tomography (SPECT). Such a platform is ideal for nanotheranostics, where therapy and diagnostics is combined in one drug delivery unit [6, 7]. An appealing objective is to further look for theranostic elements, where the electromagnetic radiation of one isotope is suitable for diagnostics and the particle radiation of another provides radiotherapy on a very local level, while both naturally exhibit the same chemical properties. Recently, scandium and the lanthanoid terbium have been investigated as promising candidates of such elements [8, 9]. For instance, in terbium, a matched isotopic multiplet of theranostically relevant radioisotopes has been suggested, where ^149^Tb and ^161^Tb provide radiotherapy together with ^152^Tb and ^155^Tb being suitable for PET and SPECT, respectively [9]. Three of these radiolanthanoids (^149^Tb, ^152^Tb and ^155^Tb) are, moreover, examples of medically suitable radionuclides that can only be produced in suitable quantities and purities at large-scale ion-beam facilities such as ISOLDE located at CERN.

The present study uses mesoporous silicon (PSi) as a carrier, which has shown very promising recent results in drug delivery studies [10–17]. PSi as a biomaterial was first suggested by L. T. Canham in 1995 based on *in vitro* studies [18], and since then, several studies on the biological interactions and resulting applications of PSi have been reviewed [19–23]. PSi is a versatile material in which the pore size and surface properties can be tailored to meet the demands of the application at hand [24, 25]. It is, in addition, biocompatible [18,26–30] and can be made biodegradable [31], and thus suitable for use in a living body as the carrier itself does not leave a trace in the tissue [31, 32]. Three types of PSi microparticles have been surface radiolabelled with ^18^F [33], the most widely used radionuclide for PET imaging. A recent study [34] also demonstrated the theranostic ability of PSi nanoparticle carriers by loading the particles with an antiangiogenic hydrophobic drug sorafenib together with a surface-conjugated ^111^In for diagnostics.

An example of a therapeutic porous silicon system is the OncoSil™ device (earlier BrachySil™) based on BioSilicon™, where the PSi is doped with stable phosphorus. The device was developed by pSiMedica Ltd UK and is currently owned by OncoSil Medical Ltd. The phosphorus, which is used as a dopant, is activated through thermal neutron capture in a nuclear reactor, and the resulting radionuclide ^32^P decays by beta emission giving a therapeutic dose to the surrounding tissue [35]. The device is used in brachytherapy where it is injected directly into an unresectable solid tumor. Clinical trials have shown good results against hepatocellular carcinoma and, moreover, compatibility with chemotherapy against pancreatic cancer. Currently pilot studies (identifiers NCT03003078 and NCT03076216) are ongoing for OncoSil™ together with standard chemotherapy. For theranostic applications, however, the disadvantage of the pure beta emitter ^32^P is its unsuitability for diagnostics as its radioactive decay does not include the emission of electromagnetic radiation.

Here, we aim to employ the radiolanthanoid ^159^Dy implanted into mesoporous silicon by a radioactive ion beam. The nuclide ^159^Dy is not commonly considered as being a good candidate for therapeutic purposes due to its long half-life of 144.4 days [36]. However, the long half-life enables the study of the stability of the theranostic system over a long time period, as the radionuclides possibly released from the PSi carrier are still radioactive. It is, therefore, an excellent model lanthanoid for the present study, where *in vivo* stability and distribution of the PSi carrier system is scrutinized. Dysprosium has, moreover, similar properties as its neighboring lanthanoid terbium. Both exhibit a characteristic oxidation state of 3^+^ [37], and their biodistribution as reported by Durbin et al. is comparable [38]. Hence, we suggest that dysprosium is a suitable analogue, for instance, for the emerging matched theranostic quadruplet of terbium.

In the decay of ^159^Dy to ^159^Tb, only one gamma ray is emitted with more than 0.1% intensity. With an energy of 58 keV [36], it is observable in gamma-ray imaging *in vivo*, but still low enough for efficient radioprotection. In addition, X-rays of 44 keV (*K*_*α*_) and 50–52 keV (*K*_*β*_) present the most intense source of electromagnetic radiation that can be used to detect and quantify the nuclide. Together with its electron-capture decay, the nucleus also emits low-energy (<58 keV) conversion electrons and Auger electrons. These electrons can produce an observable radiation dose on the surrounding tissue.

This work presents the first experiment to directly implant radionuclides obtained from ISOLDE facility to THCPSi substrates, and to process them into radionuclide-implanted THCPSi nanoparticles. The stability of the implanted ^159^Dy label is tested in an *in vivo* study in mice. The stability of the intratumorally injected theranostic particles was studied over a 17-day time period by observing noninvasively the radiation from the implanted radionuclide. Furthermore, a biodistribution study was performed to assess the site and extent of radioactivity released from the tumor.

## 2. Materials and Methods

The PSi microparticles were prepared by electrochemically anodizing a boron doped p^+^-type Si 〈100〉 wafer with a resistivity of 0.01–0.02 Ωcm in a 1 : 1 (vol.) hydrofluoric acid-ethanol electrolyte [21, 32]. The anodization current density was pulsed from 50 to 200 mA/cm^2^ sequentially, producing alternating low and high porosity layers with a thickness of ca. 200 nm and a total porous layer thickness of approximately 10 *µ*m, in order to facilitate the fragmentation of the layer into particles in postprocessing. The anodized wafer was then diced in to 15 × 15 mm^2^ substrate pieces. The surface of the fresh PSi multilayer was passivated by thermal hydrocarbonization (THCPSi), which is described in detail elsewhere [25, 33]. Briefly, the PSi substrates were placed under N_2_/C_2_H_2_ flush (1 : 1 vol.) for 15 min at room temperature followed by a thermal treatment at 500°C for 15 min. Finally, now THCPSi was allowed to cool back to room temperature under N_2_ flush.

**Figure 1:**
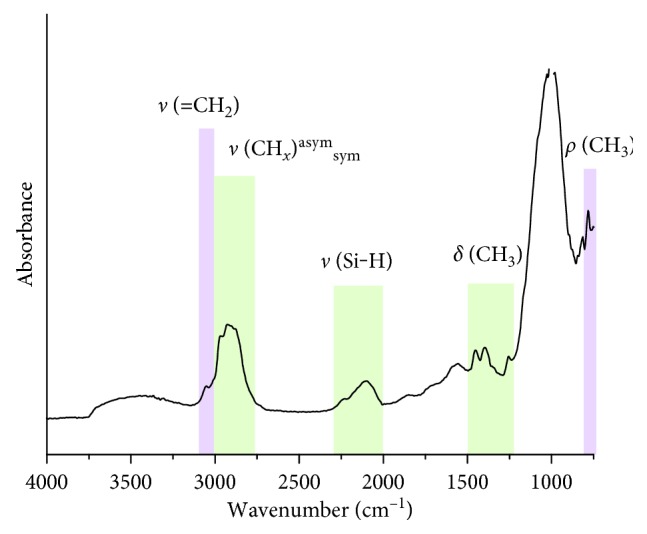
Photoacoustic FTIR spectra of the ultrasonicated THCPSi microparticles. The surface chemistry of the THCPSi microparticles was studied with a Mattson 6020 FTIR spectrometer equipped with a Gasera PA301 photoacoustic detector using 8 cm^−1^ resolution.

The surface chemistry of the THCPSi particles is shown through Fourier-transform infrared spectroscopy (FTIR) in Figure 1. The characteristic features of the thermally hydrocarbonized surface are distinctly visible. The broad band around 1000 cm^−1^ can be related to the Si-C structures [1], while the multiple bending vibrations of different CH_*x*_ groups are observable between 1200 and 1500 cm^−1^. The broad absorbance band around 2100 cm^−1^ indicates the presence of some remaining Si–H bonds from the initial fresh PSi. At 2850–2970 cm^−1^, the asymmetric and symmetric CH_2_ and CH_3_ stretching vibrations can be observed, as well as the vinyl bond = CH_2_ stretch at 3060 cm^−1^.

The THCPSi substrates were implanted at the ISOLDE ion-beam facility at CERN. A proton beam with a beam intensity of 1.5–2 *µ*A impinged on a primary tantalum target. Radioactive lanthanoid nuclides were produced in the target through nuclear spallation, thermally released from the 2000°C hot tantalum foil target, ionized in a 2000°C hot tungsten cavity, accelerated with a voltage of 30 kV, and mass-separated with the magnetic sector field of the ISOLDE general-purpose separator [39]. The thermal ionization of dysprosium was significantly enhanced with the resonance ionization laser ion source [40, 41] tuned to dysprosium, yielding a radioactive ion beam of ^159^Dy with an intensity of 10^10^ particles per second. The THCPSi substrates were mounted inside a collection chamber on the high-mass side of the general-purpose separator. The ^159^Dy ions were implanted into the mesoporous layer of the substrates with an energy of 30 keV. Four 15 × 15 mm^2^ substrates were implanted over a time of 39 h. The total activity of ^159^Dy collected in the substrates was roughly 30 MBq. In addition, the main impurity in the irradiated substrates consisted of ^143^Pm, obtained as a molecular side band from the primary target. The total activity of ^143^Pm in the substrates was roughly 2 MBq, which is close to 6% of the total activity obtained.

The substrates were transported to the University of Helsinki for postimplantation processing. The porous layers in each intact substrate were processed to microparticles through ultrasonication. The substrate was placed inside a glass vial and immersed into 99.5% ethanol with the mesoporous layer facing downward. The glass vial was pressed against the point of highest intensity on top of the ultrasonication crystal of a commercial bath sonicator and sonicated between 30 min and 2 h. After the ultrasonication, the remaining substrates were removed and the mesoporous layers, now detached from the substrate and suspended in the ethanol and were further sonicated for 3–6 h in order to reduce the size of the particles. The ethanol was then exchanged stepwise via centrifugation to a 0.9% saline solution, with a remainder of roughly 13 vol.% of ethanol in the suspension in order to keep the hydrophobic particles dispersed. After postprocessing, over 75% of the original activity was obtained in the final [^159^Dy]THCPSi suspension. Inactive reference substrates from the same batch as the radioactive substrates were prepared identically in order to characterise the structure of the THCPSi particles. The appearance of the THCPSi multilayers and the ultrasonicated particles was characterized using JEOL JSM-7500FA and Zeiss Sigma VP field-emission SEMs, while the size distribution of ultrasonicated particles was determined with laser diffraction using Sympatec HELOS equipped with a CUVETTE dispersing unit and ethanol as the dispersant.

The animal experiments described here were performed under an experimental animal license in accordance with the legal and ethical requirements as defined in the Finnish act on animal experimentation 497/2013, Finnish decree on the use of experimental animals 564/2013 (Ministry of Agriculture and Forestry), EU directive 2010/63/EU on the protection of animals used for scientific purposes, and Recommendations of the Commission of the European Communities, 2007/526/EC. During injections the animals were kept under anesthesia per inhalation of isoflurane (2% isoflurane in O_2_: medical air carrier). The animals were housed in groups of five in HEPA-filtered air flow cabinets with constant temperature (21 ± 1°C) and humidity (55 ± 10%). Lighting followed the rhythm of 12 : 12 h. The animals had access to food and water ad libitum. The well-being of the animals was monitored on a daily basis throughout the experiment, and if humane end-points were met prior to the predetermined end-point, the animal was euthanized. In the present case, the predominant end-point of this kind was the size of the tumor reaching the allowed maximum of 1.4 cm in any direction. The euthanasia of all animals was performed through asphyxiation by CO_2_ followed by cervical dislocation.

Prostate xenografts of 5 × 10^6^ PC-3MM2 cells in culture medium were subcutaneously implanted on both flanks of twenty male Hsd : NMRI-Foxn1 nude mice (7-8 weeks, Harlan), and the mice were divided into four groups (A-D), each consisting of five mice. After one week, the [^159^Dy]THCPSi suspension was injected into the tumors. In groups A and B, 20 *µ*L of [^159^Dy]THCPSi suspension was injected into each tumor. In one group (group C), 30 *µ*L was injected into each tumor. The remaining group of four mice (group D) was kept as a reference group without any injection into the tumors. The size of each tumor was measured at even time points with a digital caliper.

The activity of each tumor with injected [^159^Dy]THCPSi was measured noninvasively at even time points by using a lead-collimated hand-held NaI scintillation detector (BICRON/CANBERRA) at a distance of roughly 2 cm from the tumor. A *γ*-ray spectrum was collected from which the characteristic peaks of ^159^Dy were analyzed.

Venous blood samples were collected at designated time points into 0.3 *µ*L sized microcapillary strips. The groups of mice were euthanized at predetermined time points, so that group B was euthanized after one week, group A after two weeks, and group C after three weeks together with the reference group. Selected organs and both tumors were collected from each animal in groups A–C. Two mice in each group were rejected from the analysis due to unsuccessful intratumoral injection observed via the noninvasive gamma activity measurements. The tumors were instantly snap-frozen in isopentane cooled with dry ice and further stored at a temperature of −20°C.

The activity of all organ and blood samples was measured with a 1480 WIZARD 3” automatic gamma counter. The activity of the injection syringes and the collected tumors were measured with a Veenstra VDC-405 V3.30 dose calibrator. Additionally, the blood samples taken at one and three days after injection were measured with a Canberra thin-entry window GX8021 high-purity germanium (HPGe) detector using the 44-keV X-ray doublet and the 58-keV *γ*-ray peak, both originating from ^159^Dy. Each tumor was cryosectioned into a thickness of 30 *µ*m with a Leica CM 1950 cryostat collecting every other section for autoradiography and every other for hematoxylin and eosin (H&E) staining for anatomical reference. Autoradiographic images were obtained for the unstained sections with a Beaver micropattern gas detector (MPGD) detector system [42, 43].

## 3. Results

**Figure 2:**
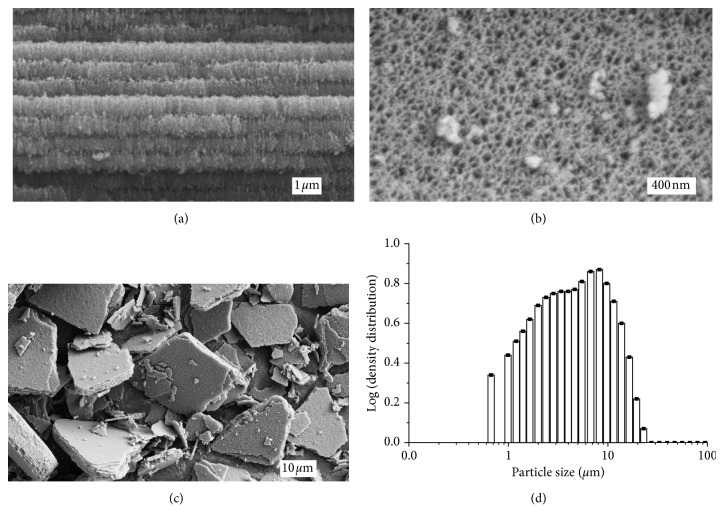
(a)–(c) FESEM images produced of the mesoporous THCPSi structure of the inactive reference substrates. (a) A cross-sectional view of the layer-like structure of the mesoporous surface. Each layer has an approximate thickness of 200 nm. (b) Image taken from above the structure depicts the inlets of the pores from the top. (c) Image produced of the mesoporous THCPSi particle distribution after ultrasonication. (d) Size distribution of the THCPSi reference particles obtained by laser diffraction.

Inactive reference substrates from the same batch as the radioactive substrates were prepared identically in order to characterize the structure of the THCPSi particles.  Figure 2(a) presents a field-emission scanning electron microscope (FESEM) image of a cross section of the THCPSi substrate surface region prior to ultrasonication, showing the alternating low/high-porosity regions due to the use of pulsed etching current profile. The pore openings can be seen in the top view of the structure presented if Figure 2(b), showing the layer being completely mesoporous. As the surface is exposed to ultrasonication, the layers are fractured into particles, partially according to the premade high porosity fracture planes, but mainly into thicker microparticles.  Figure 2(c) presents the particle size distribution visualised by FESEM. The particle size distribution according to laser diffraction measurements in EtOH dispersion shown in Figure 2(d) confirms the [^159^Dy]THCPSi particles as small microparticles, with a *d*_90_ value of 12.5 *µ*m and a volume mean diameter of 5.5 *µ*m. As in this method, the device optics limits the data to above 0.5 *µ*m, and the presence of THCPSi nanoparticles in the dispersion was not verified.

The injected suspension had an activity concentration of 11.5 ± 0.6 kBq/*µ*l. The average injected activity per mouse in groups A and B was 136 ± 13 kBq (excluding one clearly failed injection) and in group C 330 ± 70 kBq. A variation in activities was due to a quite significant amount of activity staying in the syringe after injection (66 ± 11% of activity). This suggests a problem in formulation, probably due to a large variation in particle sizes, since clearly part of the particles is not properly suspended but sticks on the surfaces of the syringe.

**Figure 3:**
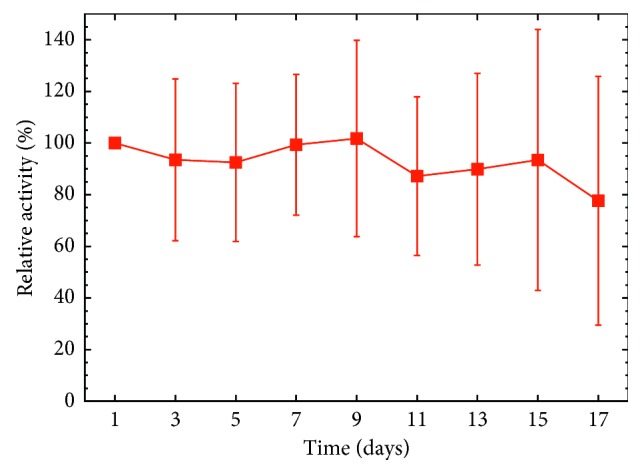
Average relative activities of tumors with reference to the first data point taken one day after injection of [^159^Dy]THCPSi. The uncertainties are taken as one standard deviation. The measurements were performed in vivo with a hand-held NaI detector; see text in Chapter 3 for details.

**Figure 4:**
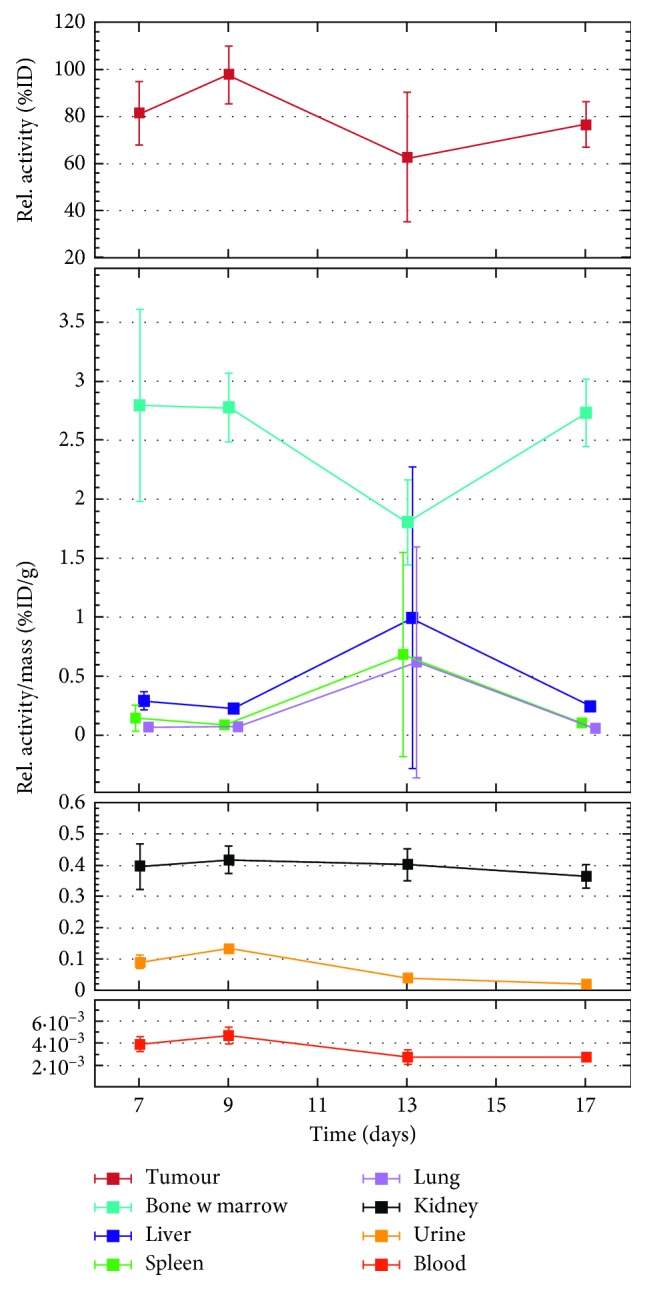
Biodistribution of ^159^Dy in selected organs as a function of time. The activity has been normalized to the total injected activity and to the weight of the organ sample (except for the uppermost panel depicting the tumor data, where the tumor weight has been omitted). The uncertainties have been taken as one standard deviation. The data points have been shifted slightly around the value on the time axis for visual purposes. The data consist of a varying number of animals: *n*=4 (7 days), *n*=1 (9 days), *n*=3 (13 days), and *n*=1 (17 days). The large deviation in the systematic trend at thirteen days originates from one animal. When *n*=1, the uncertainty was taken directly as directly as the propagated error in the measurement.

The activity of each tumor was measured *in vivo* at designated time points, to probe the possible changes in activity indicating a release of the radionuclide. The first data point was taken as a reference value, and all subsequent values were normalized to it. An average relative activity over all the animals in each group was then calculated.  Figure 3 presents the results from the *in vivo* tumor activity measurements. It can be seen from the figure that the activity stays stable over the first week after which it starts gradually to decrease down to eighty percent at seventeen days. The uppermost panel in Figure 4 presents the *ex vivo* activity of the tumors as normalized to the injected activity. The values stay quite stable throughout the measurement period, indicating that the activity stays stable inside the tumors.

Selected organs were collected from the euthanized animals at 7, 9, 13, and 17 days after particle administration. A biodistribution study was performed on the organs to observe where the activity possibly escaping from the tumor is accumulated. Figure 4 presents the biodistribution of ^159^Dy as a function of time after injection. The time points at 9 and 13 days have only one animal. In such a case, a statistical analysis of the uncertainties of the results is not feasible. The best estimate for the uncertainty is then obtained simply from the error of the radioactivity measurement together with the accuracy of the sample weight. These uncertainties are then propagated to the final result. For more details, see, for instance, the handbook by Taylor [44]. The bone uptake is moderately high at 2.8 (9) %ID/g already at the first time point at 7 days, and the behavior of the activity stays very stable over time without an observable increase. Although the bone uptake is moderately high, the total amount of ^159^Dy injected into the animal is in the region of tens of ppb, indicating the sensitivity of gamma-ray spectrometry as a probe of particle concentration.

**Figure 5:**
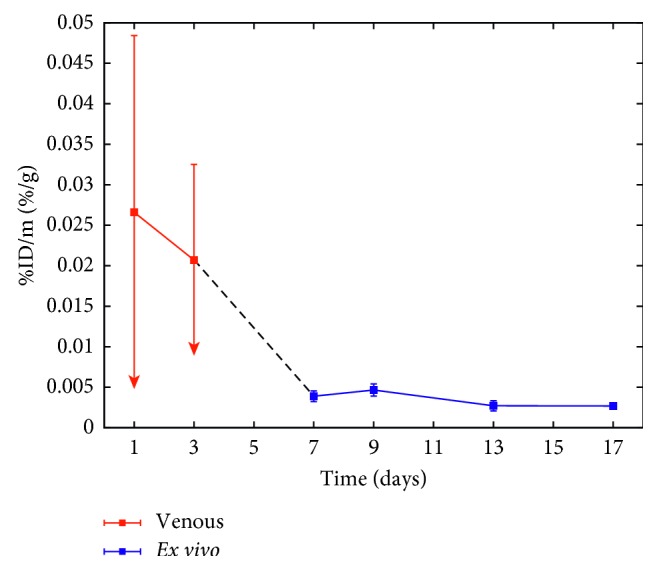
Combined results of activity in the venous blood (days 1 and 3 indicated in red) and *ex vivo* (day 7 and further indicated in blue). The data obtained from the venous blood present upper limits of the activity as part of the samples lay beneath the MDA of the HPGe detector, in which case the MDA was used.

Venous blood samples were collected at even time points to observe the possible leak of the activity into the blood circulation. The activity of the samples was measured, and all data points lay beneath the nuclide-specific minimum detectable activity (MDA) [45] of 0.1 Bq of the WIZARD counter. To demonstrate the low activity of the blood samples, the first and second data points, taken one and three days, respectively, after the injection of the [^159^Dy]THCPSi into the tumor were remeasured with a thin-entry-window HPGe detector to gain accuracy in activity.  Figure 5 presents these results combined with the corresponding results obtained *ex vivo*. For the first two data points, a burst in activity is observed in the data, after which the activity in the blood is reduced.

**Figure 6:**
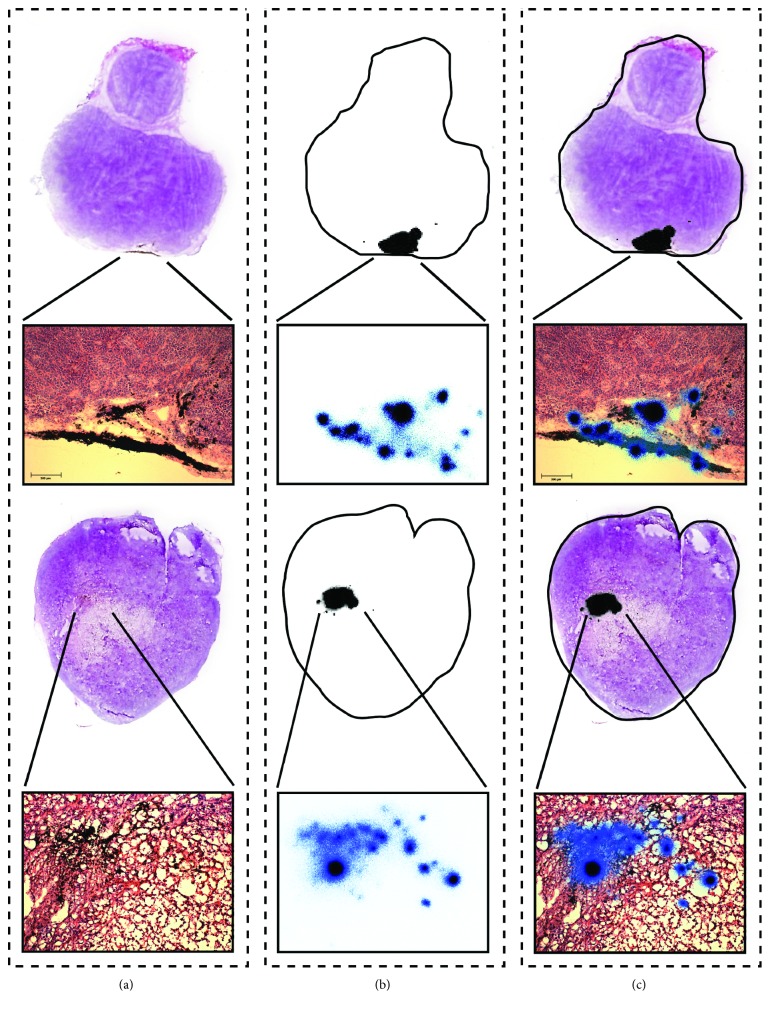
Combined histological and autoradiographic data from two example tumors. (a) Photographed histological section stained with hematoxylin and eosin, together with a microscope image of the relevant region produced with a tenfold magnification. The dark areas in the microscope image present the injected [^159^Dy]THCPSi particles. (b) Corresponding autoradiographic image produced to the neighboring unstained section together with the outline of the section. The gross properties of the activity is presented with a 50 *µ*m resolution and the detailed properties in the magnified region with a 2 *µ*m resolution. Both images were obtained from the same measurement. (c) Combined information of (a) and (b). The upper row presents a tumor with the [^159^Dy]THCPSi particles present close to the surface and the bottom row a tumor with the particles closer to the center.

Although the primary aim of our experiment was to investigate the biodistribution and *in vivo* stability of the [^159^Dy]THCPSi and not their therapeutic effect, the growth of the tumors was studied over time and compared between groups A–D. No observable effect on the size was detected when comparing the groups. More local effects were assessed at a cellular scale by studying the stained histological samples together with the results from the autoradiographic study, but no clear indications of cellular damage could be obtained.  Figure 6 presents the results of the histological and autoradiographic study for two example tumors. The radioactive region indicated in black is very localized inside the histological section. In a more detailed microscope image, the radioactive region is shown to correspond well to the THCPSi particle distribution visible in a dark color on the microscope image.

## 4. Discussion

The results from the in vivo tumor activity show, although the uncertainties are quite significant, that a temporal trend can be observed. This trend indicates that the activity stays stable in the tumor until day 9 of the observation period. The main source of uncertainty is likely caused by that of the measurement geometry as the radiation source did not remain completely stationary throughout the measurement. The *in vivo* results are, furthermore, supported by those obtained from the *ex vivo* activity measurements performed for the tumors collected at the end point, showing a relatively stable activity of 80% of injected activity over the observation period.

Based on the results from the biodistribution, it is evident that the highest accumulation of free ^159^Dy is in the bone. Such a route is expected for a heavy lanthanoid based on the study by Durbin et al. where it was shown that a larger atomic number of the lanthanoid corresponds to a higher fraction of the accumulation into the bone [38]. The accumulation into the bone is temporally stable, albeit moderately high. We, therefore, suggest that the activity accumulated in the bone originates from free ^159^Dy in an initial burst released immediately after the injection of the particles. This is supported by the fact that the activity of the *ex vivo* blood samples in Figures 4 and 5 stays at a very low level throughout the observation period, while the venous blood in Figure 5 has a higher level. Moreover, we did not observe any elimination of ^159^Dy into the urine at later time points, which together with the low blood activity levels supports our suggestion that the activity accumulated in the bone originates dominantly from initially free ^159^Dy in the particle formulation and not from a gradual release from the THCPSi. The burst likely consists from free ^159^Dy that has dissolved in the saline solution during storage, as the [^159^Dy]THCPSi was prepared four days before injection. The ^159^Dy atoms are confined in the THCPSi crystal structure during implantation. Therefore, a disintegration of the THCPSi structure would free the ^159^Dy atoms, and the activity would be observed in the blood circulation. Thus, we show not only that the THCPSi particles stay intact in the tumor but also that the implanted ^159^Dy remains embedded in the THCPSi crystal structure.

The absorbed dose obtained in the tumor during the observation period was estimated to be in the order of 1 Gy, which is two orders of magnitude lower than the recommended permanent prostate brachytherapy (PPB) dose of over 100 Gy for a similar radionuclide [46]. Therefore, a macroscopically observable therapeutic effect on the tumor size cannot be expected. Effects on a microscopic level were studied but could not be confirmed. The low injected activity together with the short range of the emitted electrons results in the radiation causing no therapeutic effect on the tumor as can be expected.

The THCPSi particles stay very localized inside the tumor as can be seen in Figure 6. The reason for this remained unclear, but it could originate from the large particle size. The locality, however, enables the observation that no activity is detected outside the particle distribution inside the tumor.

As our initial results show a good stability in the tumor using our model radionuclide, future studies will include the usage of therapeutically attractive RIB-based radiolanthanoids to be able to observe a macroscopically visible therapeutic effect on the tumor, with a suitable half-life to ensure the decay of the nuclide before the dissolution of the THCPSi carrier starts. Moreover, to reduce the amount of free radionuclide prior to injection, the postprocessing procedures and formulation of the final injectable suspension require further optimization. The surface chemistry of THCPSi makes the PSi particles durable, but very hydrophobic, resulting in agglomeration. In order to obtain an even activity distribution, a dispersant or a suitable coating of the particles can to be applied. Finally, for the particle to be able to penetrate the cell barrier in targeted intravenous administration, the particle size needs to be reduced down to sub-micrometer scale. This can be reached by implanting the nuclides on self-supporting porous silicon layers detached from the wafer and using high-speed milling combined with ultrasonication for particle processing.

## 5. Conclusions

The implantation of radionuclides into THCPSi by a radioactive ion beam and its further processing to radionuclide-implanted particles have been studied and shown to produce a stable system for radiation theranostics. Postprocessing of the radioactive THCPSi by sonication does, however, not produce a sufficiently sharp size distribution in a case such as the present, where a high yield of the activity is pursued. The *in vivo* evaluation of the intratumorally injected [^159^Dy]THCPSi indicates that the particles and their radioactive label stay stable and close to the injection point inside the tumor during the 17-day observation time. Since the released ^159^Dy mostly accumulates into the bone, a stable bond to the carrier until its radioactive decay is essential for therapeutic purposes. To conclude, we suggest that THCPSi is suitable for use as a carrier for radiation theranostics together with a RIB-based radiolanthanoid, which opens wide possibilities for theranostic applications due to the large library of nuclides available for implantation and the possibilities provided by the porous silicon platform. Furthermore, the crystal structure of Si and a vast knowledge of ion-implantation in it would enable many different possibilities to extend the usability of the method in future. None of any other delivery agents can offer this kind of versatility.

## Data Availability

The data in the form of graphs/images used to support the findings of this study are included within the article. The numerical data of what the figures/images are based on are available from the corresponding author upon request.

## References

[1] http://www.who.int/cancer/en

[2] Sawyers C. (2004). Targeted cancer therapy. *Nature*.

[3] Hamoudeh M., Kamleh M. A., Diab R., Fessi H. (2008). Radionuclides delivery systems for nuclear imaging and radiotherapy of cancer. *Advanced Drug Delivery Reviews*.

[4] Sanvicens N., Marco M. P. (2008). Multifunctional nanoparticles-properties and prospects for their use in human medicine. *Trends in Biotechnology*.

[5] Torchilin V. P. (2012). Multifunctional nanocarriers. *Advanced Drug Delivery Reviews*.

[6] Choi K. Y., Liu G., Lee S., Chen X. (2012). Theranostic nanoplatforms for simultaneous cancer imaging and therapy: current approaches and future perspectives. *Nanoscale*.

[7] Kelkar S. S., Reineke T. M. (2011). Theranostics: combining imaging and therapy. *Bioconjugate Chemistry*.

[8] Müller C., Bunka M., Haller S. (2014). Promising prospects for ^44^Sc-/^47^Sc-based theragnostics: application of 47Sc for radionuclide tumor therapy in mice. *Journal of Nuclear Medicine*.

[9] Müller C., Zhernosekov K., Köster U. (2012). A unique matched quadruplet of terbium radioisotopes for PET and SPECT and for-and-radionuclide therapy: an in vivo proof-of-concept study with a new receptor-targeted folate derivative. *Journal of Nuclear Medicine*.

[10] Tasciotti E., Liu X., Bhavane R. (2008). Mesoporous silicon particles as a multistage delivery system for imaging and therapeutic applications. *Nature Nanotechnology*.

[11] Foraker A. B., Walczak R. J., Cohen M. H., Boiarski T. A., Grove C. F., Swaan P. W. (2003). Microfabricated porous silicon particles enhance paracellular delivery of insulin across intestinal caco-2 cell monolayers. *Pharmaceutical Research*.

[12] McInnes S. J. P., Turner C. T., Al-Bataineh S. A. (2015). Surface engineering of porous silicon to optimise therapeutic antibody loading and release. *Journal of Materials Chemistry B*.

[13] Ferreira M. P. A., Ranjan S., Kinnunen S. (2017). Drug-loaded multifunctional nanoparticles targeted to the endocardial layer of the injured heart modulate hypertrophic signaling. *Small*.

[14] Blanco E., Sangai T., Hsiao A. (2013). Multistage delivery of chemotherapeutic nanoparticles for breast cancer treatment. *Cancer Letters*.

[15] Kinnari P. J., Hyvönen M. L. K., Mäkilä E. M. (2013). Tumour homing peptide-functionalized porous silicon nanovectors for cancer therapy. *Biomaterials*.

[16] Xu W., Thapa R., Liu D. (2015). Smart porous silicon nanoparticles with polymeric coatings for sequential combination therapy. *Molecular Pharmaceutics*.

[17] Tanaka T., Mangala L. S., Vivas-Mejia P. E. (2010). Sustained small interfering RNA delivery by mesoporous silicon particles. *Cancer Research*.

[18] Canham L. T. (1995). Bioactive silicon structure fabrication through nanoetching techniques. *Advanced Materials*.

[19] Santos H. A., Mäkilä E., Airaksinen A. J., Bimbo L. M., Hirvonen J. (2014). Porous silicon nanoparticles for nanomedicine: preparation and biomedical applications. *Nanomedicine*.

[20] Anglin E., Cheng L., Freeman W., Sailor M. (2009). Porous silicon in drug delivery devices and materials. *Advanced Drug Delivery Reviews*.

[21] Salonen J., Kaukonen A. M., Hirvonen J., Lehto V. P. (2008). Mesoporous silicon in drug delivery applications. *Journal of Pharmaceutical Sciences*.

[22] Santos H. A. (2014). *Porous Silicon for Biomedical Applications*.

[23] Canham L. (2014). *Handbook of Porous Silicon*.

[24] Salonen J., Lehto V. P. (2008). Fabrication and chemical surface modification of mesoporous silicon for biomedical applications. *Chemical Engineering Journal*.

[25] Salonen J., Laine E., Niinistö L. (2002). Thermal carbonization of porous silicon surface by acetylene. *Journal of Applied Physics*.

[26] Bayliss S. C., Heald R., Fletcher D. I., Buckberry L. D. (1999). The culture of mammalian cells on nanostructured silicon. *Advanced Materials*.

[27] Low S. P., Voelcker N. H., Canham L. T., Williams K. A. (2009). The biocompatibility of porous silicon in tissues of the eye. *Biomaterials*.

[28] Tanaka T., Godin B., Bhavane R. (2010). In vivo evaluation of safety of nanoporous silicon carriers following single and multiple dose intravenous administrations in mice. *International Journal of Pharmaceutics*.

[29] Näkki S., Rytkönen J., Nissinen T. (2015). Improved stability and biocompatibility of nanostructured silicon drug carrier for intravenous administration. *Acta Biomaterialia*.

[30] Tölli M. A., Ferreira M. P. A., Kinnunen S. M. (2014). In vivo biocompatibility of porous silicon biomaterials for drug delivery to the heart. *Biomaterials*.

[31] Park J. H., Gu L., von Maltzahn G., Ruoslahti E., Bhatia S. N., Sailor M. J. (2009). Biodegradable luminescent porous silicon nanoparticles for in vivo applications. *Nature Materials*.

[32] Bimbo L. M., Sarparanta M., Santos H. A. (2010). Biocompatibility of thermally hydrocarbonized porous silicon nanoparticles and their biodistribution in rats. *ACS Nano*.

[33] Sarparanta M., Mäkilä E., Heikkilä T. (2011). ^18^F-labeled modified porous silicon particles for investigation of drug delivery carrier distribution in vivo with positron emission tomography. *Molecular Pharmaceutics*.

[34] Wang C. F., Sarparanta M. P., Mäkilä E. M. (2015). Multifunctional porous silicon nanoparticles for cancer theranostics. *Biomaterials*.

[35] Zhang K., Loong S. L. E., Connor S. (2005). Complete tumor response following intratumoral 32P BioSilicon on human hepatocellular and pancreatic carcinoma xenografts in nude mice. *Clinical Cancer Research*.

[36] Helmer R. G. (2003). Nuclear data sheets for A = 159. *Nuclear Data Sheets*.

[37] Moeller T. (1963). *The Chemistry of the Lanthanides*.

[38] Durbin P. W., Williams M. H., Gee M., Newman R. H., Hamilton J. G. (1956). Metabolism of the lanthanons in the rat. *Experimental Biology and Medicine*.

[39] Kugler E., Fiander D., Johnson B. (1992). The new CERN-ISOLDE on-line mass-separator facility at the PS-Booster. *Nuclear Instruments and Methods in Physics Research Section B: Beam Interactions with Materials and Atoms*.

[40] Köster U., Fedoseyev V. N., Mishin V. I. (2003). Resonant laser ionization of radioactive atoms. *Spectrochimica Acta Part B: Atomic Spectroscopy*.

[41] Rothe S., Marsh B. A., Mattolat C., Fedosseev V. N., Wendt K. (2011). A complementary laser system for ISOLDE RILIS. *Journal of Physics: Conference Series*.

[42] Donnard J., Thers D., Servagent N., Luquin L. (2009). High spatial resolution in-imaging with a PIM device. *IEEE Transactions on Nuclear Science*.

[43] Donnard J., Arlicot N., Berny R. (2009). Advancements of labelled radio-pharmaceutics imaging with the PIM-MPGD. *Journal of Instrumentation*.

[44] Taylor J. R. (1997). *An Introduction to Error Analysis*.

[45] Currie L. A. (1968). Limits for qualitative detection and quantitative determination. Application to radiochemistry. *Analytical Chemistry*.

[46] Davis B. J., Horwitz E. M., Lee W. R. (2012). American brachytherapy society consensus guidelines for transrectal ultrasound-guided permanent prostate brachytherapy. *Brachytherapy*.

